# Association of the growth hormone c.2141C>G polymorphism with growth performance, slaughter traits, and meat quality in Kalmyk bull calves

**DOI:** 10.14202/vetworld.2026.3961-3975

**Published:** 2026-09-11

**Authors:** Nadezhda Chimidova, Natalia Revutskaya, Anastasia Semenova, Victoria Ubushieva, Vasilii Manzhiev, Arslang Khakhlinov, Altana Ubushieva, Zanda Bochkaeva

**Affiliations:** 1 Regional Center of Science and Production, Kalmyk State University named after B.B. Gorodovikov, 11, Pushkin str., 358000, Elista, Republic of Kalmykia, Russian Federation.; 2 Department of Scientific, Applied and Technological Developments, V.M. Gorbatov Federal Research Center for Food Systems of RAS, 26, Talalilhin str., 109316, Moscow, Russian Federation.

**Keywords:** beef cattle genetics, beef quality, carcass characteristics, growth hormone gene, growth performance, Kalmyk cattle, marker-assisted selection, meat productivity

## Abstract

**Background and Aim:**

The growth hormone (*GH*) gene is an important candidate locus for marker-assisted selection because its polymorphisms may influence growth, carcass characteristics, and beef quality. However, evidence linking the *GH* c.2141C>G polymorphism with meat productivity and physicochemical characteristics in Kalmyk cattle remains limited. This study aimed to evaluate the association of the *GH* c.2141C>G polymorphism with growth performance, slaughter traits, and beef quality in Kalmyk bull calves.

**Materials and Methods:**

Fifty Kalmyk bull calves were genotyped for the *GH* c.2141C>G polymorphism and classified as LL (n = 37) or LV (n = 13). Animals were monitored from 8 to 15 months of age under standardized feeding and housing conditions. Live weight and average daily gain were evaluated during the growth period. At 15 months, eight animals from each genotype-group were selected for slaughter and assessment of carcass characteristics and beef chemical composition. Genotype-associated differences were evaluated using appropriate statistical tests with correction for multiple comparisons, and Cohen’s d was used to assess effect magnitude.

**Results:**

LV bull calves had higher live weights than LL calves by 4.45, 20.5, and 25.9 kg at 8, 12, and 15 months, respectively, and their average daily gain was 5.1%–11.5% higher across the evaluated periods. However, these differences were not statistically significant (p > 0.05), although effect sizes indicated potentially relevant differences in growth performance. Significant genotype-associated differences were observed in slaughter and meat-quality traits. Carcass weight and internal fat weight showed large effect sizes (Cohen’s d = 2.28 and 3.53, respectively). Beef chemical composition also differed significantly between genotypes (p = 0.001–0.0013; Cohen’s d = 1.25–1.47). The LV genotype was associated with 1.01% higher protein and 0.23% higher ash contents, whereas the LL genotype was associated with 0.5% higher fat and 0.4% higher moisture contents.

**Conclusion:**

The *GH* c.2141C>G polymorphism was associated more strongly with slaughter characteristics and beef chemical composition than with growth performance in Kalmyk bull calves. The LV genotype showed favorable trends in growth and was associated with higher protein and ash contents, supporting further evaluation of this polymorphism as a potential marker for selection programs targeting meat productivity and quality. Larger populations are required to confirm its utility for marker-assisted selection.

## INTRODUCTION

Global beef cattle statistics as of late 2025 indicate an overall shortage in cattle numbers and a constrained global beef market [1]. At the same time, the global market is characterized by increasing competition in premium beef segments (Prime and Choice), with greater emphasis on marbling, tenderness, and organoleptic properties; increasing demand for products with verified genetic origin and environmental sustainability, such as Certified Angus Beef and Grass Fed Beef certification; and the implementation of genomic selection programs, including the GeneSeek Genomic Profiler (Angus GGP) program in the United States, Cooperative Research Centre for Beef Genetic Technologies (Beef CRC) in Australia, and EuroGenomics in the European Union, to accelerate genetic progress in meat productivity traits [2, 3]. Numerous national and international studies have demonstrated the potential of molecular genetic markers to predict and selectively improve economically important meat productivity traits [4–7]. Traditional phenotypic assessment methods have several limitations, including high labor requirements, prolonged evaluation periods, and dependence on housing and management conditions [8]. Consequently, identifying single-nucleotide polymorphisms (SNPs) associated with quantitative and qualitative meat traits has become increasingly relevant [9–12].

One of the major genes investigated for marker-assisted selection of economically important traits in beef cattle is the growth hormone (*GH*) gene, located on bovine chromosome 19 (BTA19) and comprising five exons and four introns [13]. One of the most extensively studied *GH* polymorphisms is c.2141C>G (exon 5, 19:48118256C>G, rs41923484, L127V), which has been associated with variability in growth and development and with quantitative and qualitative meat productivity traits [13–15]. In the literature, the c.2141C>G polymorphism is also described as L/V, denoting a leucine-to-valine amino acid substitution [16]. This missense mutation alters the structure of the somatotropin protein and may affect the growth regulation and muscle tissue development [17]. Associations between the *GH* c.2141C>G polymorphism and slaughter traits in cattle have been reported [18–21]. A previous study also indicated an association between this polymorphism and the amino acid composition of meat [22].

With the intensification of livestock production and increasingly stringent food safety requirements, assessment of the physicochemical characteristics of beef has become increasingly important. Integrating physicochemical evaluation with marker-assisted selection may contribute to sustainable beef production by combining desirable consumer characteristics, food safety, and economic efficiency [23, 24].

In Russia, the beef cattle industry has increasingly emphasized specialized beef breeds, including Angus, Hereford, and Kalmyk cattle. Their reported share increased from 12% in 2010 to 28% in 2024 as part of efforts toward import substitution and expansion of export capacity, with a target of 500,000 head by 2030 [25]. Concurrently, government initiatives are strengthening the implementation of genomic selection in the industry.

The Kalmyk breed (*Bos taurus turano-mongolicus*), the only native Russian beef breed, possesses several adaptive and productive characteristics that distinguish it from foreign beef breeds. These include tolerance to temperatures ranging from −30°C to +45°C, drought resistance, efficient utilization of low-productivity pastures and plant by-products such as straw, chaff, and grain residues, a high dressing percentage, and marbling scores of 3–4 points on the United States Department of Agriculture scale under feedlot conditions [26–31].

Despite increasing application of molecular markers in cattle breeding, the molecular genetic basis of economically important production traits in Kalmyk cattle remains insufficiently characterized. In particular, available evidence is inadequate to determine whether variation at the *GH* c.2141C>G locus is consistently associated with economically relevant phenotypes in this breed. This represents an important breed-specific knowledge gap because associations identified in other cattle populations may not necessarily reflect those in Kalmyk cattle, given differences in genetic background and production conditions. Furthermore, the available literature does not provide sufficient integrated evidence linking this polymorphism simultaneously with longitudinal growth performance, slaughter characteristics, and physicochemical properties of beef in Kalmyk bull calves.

Another important methodological gap concerns evaluating the magnitude and practical relevance of genotype-associated differences. Reliance exclusively on conventional statistical significance may provide an incomplete assessment, particularly when naturally occurring genotype frequencies result in unequal group sizes. An analytical approach that incorporates multiple-comparison correction and effect-size estimation is therefore needed to distinguish statistical evidence from the magnitude of observed genotype-associated differences. Addressing these gaps through an integrated evaluation of growth, slaughter, and meat-quality traits could provide a stronger basis for determining the potential utility of the *GH* c.2141C>G polymorphism in genetic evaluation and marker-assisted selection of Kalmyk cattle.

This study aimed to evaluate the association of the *GH* c.2141C>G polymorphism with growth performance, slaughter traits, and beef quality in Kalmyk bull calves under standardized rearing conditions. Specifically, the study compared live weight dynamics at different ages and average daily gain across defined growth periods between the LL and LV genotypes; evaluated slaughter traits, including carcass weight, internal fat weight, and dressing percentage; and determined genotype-associated differences in the chemical composition of beef, including moisture, protein, fat, and ash contents. In addition, statistical analyses accounting for multiple comparisons and effect-size estimates were applied to assess both the statistical evidence and practical magnitude of the observed genotype-associated differences. This integrated approach was intended to provide evidence on the potential applicability of the *GH* c.2141C>G polymorphism as a molecular marker for selecting economically important traits in Kalmyk beef cattle.

## MATERIALS AND METHODS

### Ethical approval

All procedures involving animals were reviewed and approved by the Ethics Committee for Animal Experiments of the Narmaev Kalmyk Research Institute of Agriculture (Approval No. 2, October 2, 2024). The experimental protocol covered animal selection, housing and management, clinical examination, blood collection, genotyping, growth monitoring, and subsequent slaughter and sample collection for meat-quality assessment. Throughout the study, animal handling and experimental procedures were conducted with consideration for animal welfare and with measures to minimize unnecessary stress and discomfort.

Before enrollment, all bull calves underwent clinical examination to confirm their health status and were vaccinated according to the recommended schedule. Blood samples were collected by trained personnel through jugular venipuncture under standard farm conditions, with approximately 10 mL collected per sampling session. The sampling volume and schedule were selected to meet the analytical requirements of the study while minimizing the burden on the animals. Following blood collection, the animals were monitored for adverse reactions and provided appropriate care when required. No invasive surgical procedures were performed during the experiment.

During the 7-month experimental period, the animals were maintained under standardized husbandry conditions, with access to appropriate feed and drinking water and routine monitoring of their health and welfare. Husbandry and handling procedures were performed by trained personnel to minimize stress. Slaughter of the selected animals was performed at 15 months of age in accordance with the standard meat industry procedure GOST 34120-2017 and using procedures intended to ensure humane treatment. All animals completed the experimental protocol, and no complications affecting animal welfare or study data were reported.

### Study period and location

The study was conducted from November 1, 2024, to June 1, 2025, at the evaluation station of the Regional Research and Production Center for Livestock Reproduction, Kalmyk State University named after B.B. Gorodovikov, Elista, Republic of Kalmykia, Russia. The evaluation station is used for comprehensive assessment of breed and production traits in farm animals through breeding and genetic analysis. Throughout the experimental period, the bull calves were housed and managed in accordance with the applicable requirements of the Russian Ministry of Agriculture.

### Selection criteria and sampling strategy

The study included 50 Kalmyk bull calves monitored from 8 to 15 months of age over a 7-month period. The inclusion criteria were established before the study. Eligible animals were Kalmyk bull calves at the post-weaning stage, 8 months of age, and weighing 190–200 kg at enrollment to minimize baseline variability. Until 8 months of age, the calves were reared under the cow-calf production system typical of Kalmyk beef cattle. Only animals without clinical signs of infection, injury, or chronic disease were included. All calves originated from breeding farms in the Republic of Kalmykia and had confirmed pedigrees documented by breeding certificates. Before enrollment, all animals (n = 50) were vaccinated according to the recommended schedule and underwent clinical examination to confirm their health status.

Blood samples were collected by trained personnel through jugular venipuncture under standard farm conditions. Approximately 10 mL of blood was collected per sampling session, with the volume and sampling schedule selected to meet the requirements of the study while minimizing the burden on the animals. After each blood collection, the animals were monitored for adverse reactions and provided appropriate care as needed. No invasive surgical procedures were performed.

Genotyping of the *GH* c.2141C>G polymorphism was performed using Sanger sequencing. Based on the genotyping results, 37 animals had the LL genotype, and 13 had the LV genotype. Eight bull calves from each genotype were selected for meat-quality assessment, with selection accounting for the mean live weight within each genotype-group to minimize intergroup variation. The sample size of eight animals per genotype was determined a priori based on an anticipated effect size of Cohen's d > 0.8, statistical power of 0.80, and significance level of α_adj = 0.05.

### Study design and animal housing

Before the experiment, all 50 Kalmyk bull calves were obtained from five breeding farms in the Republic of Kalmykia. The animals were vaccinated according to the recommended schedule and clinically examined to confirm their health status. Following genotyping, 37 animals were identified as LL and 13 as LV. At the university evaluation station, the animals were housed in two sections, with 25 animals in each section. Allocation to sections was performed using stratified randomization according to the farm of origin; genotype was not considered during allocation. Consequently, animals with different genotypes were co-housed within each section rather than separated into genotype-specific groups. Baseline age and live weight did not differ significantly between the sections (p > 0.05), supporting their comparability.

All animals were maintained under standardized housing and management conditions. Each section had an area of 75 m² and consisted of well-ventilated pens with straw bedding that was cleaned daily. Feed and water were provided *ad libitum*, and trained personnel performed husbandry and feeding procedures to minimize animal stress. Animals were randomly allocated to pens, and their pen locations remained unchanged between measurement periods. No buffer zones were maintained between groups and contact through pen partitions was possible. Microenvironmental factors, including proximity to feeders and ventilation openings, were not specifically controlled.

Baseline data recorded for each animal included species, sex, age, live weight, farm of origin, and clinical examination findings. Live weight was measured at 8, 12, and 15 months of age, and these measurements were used to calculate average daily gain. Feed and water intake were also recorded. All measurements were performed within a standardized time window from 8:00 to 11:00 AM.

For meat-quality assessment, eight bull calves were selected from each genotype group, for a total sample of 16 animals. Selection accounted for mean live weight within each genotype group to reduce intergroup variability and improve the sensitivity of statistical comparisons. The sample size was determined a priori based on an expected effect size of Cohen's d > 0.8, statistical power of 0.80, and significance level of α_adj = 0.05.

Slaughter was performed at 15 months of age in accordance with standard meat industry procedures (GOST 34120-2017) using methods intended to ensure humane treatment of the animals. Before slaughter, the selected calves (n = 16) underwent a 15-h lairage period, with water withheld during the final 3 h. Following slaughter, meat-quality characteristics were comprehensively evaluated, including morphological assessment of the carcass. Muscle samples intended for chemical analysis were submitted to the V.M. Gorbatov Federal Research Center for Food Systems of the Russian Academy of Sciences according to standardized protocols. All animals completed the experimental protocol, all measurements were included in the analysis, and no complications affecting data quality occurred during the study.

### Diet, feeding, and drinking schedule

All bull calves received the same diet throughout the experimental period. The diet consisted of mixed-grass hay provided at 4 kg/animal/day and compound feed comprising ground wheat, barley, corn, wheat meal, and a protein-mineral-vitamin complex. Compound feed was supplied using a bunker-type feeding system that provided *ad libitum* access to the feeders. Drinking water was also available *ad libitum* throughout the study.

### Genotyping

DNA was extracted using an automated procedure with the MagnoPrime VET commercial reagent kit (NextBio LLC, Moscow, Russia). The quality of the extracted DNA was assessed using a Nano-500 spectrophoto-meter. Sanger sequencing was used to determine the *GH* c.2141C>G genotype.

The sequencing workflow comprised five principal steps. First, polymerase chain reaction (PCR) amplification was performed using the specific oligonucleotide primers 5′-TAGGGGAGGGTGGAAAATGGA-3′ (forward) and 5′-GACACCTACTCAGACAATGCG-3′ (reverse). Second, the PCR products were purified using the ColGen kit (Syntol, Moscow, Russia), and the concentrations of the purified products were determined using a Nano-500 spectrophotometer, ranging from 49.2 to 69.5 ng/µL. Third, Sanger sequencing was performed using the GenSeq Sanger DNA sequencing reagent kit (Syntol). Fourth, the sequencing products were purified using SeqMAG (Syntol). Finally, capillary electrophoresis was performed using a Nanofor 05 genetic analyzer with PDMA-6 sequencing polymer and 1× TAPS sequencing buffer (Syntol).

The PCR reaction mixture had a total volume of 25 µL and was prepared according to the manufacturer's recommendations for Taq DNA polymerase (Evrogen, Moscow, Russia). The mixture contained 2.5 µL of 10× buffer, 1 µL of forward primer (10 µM), 1 µL of reverse primer (10 µM), 1.25 µL of deoxyribonucleotide triphosphate mixture (4 mM), 2 µL of DNA, and 0.5 µL of Taq DNA polymerase, with deionized water added to a final volume of 25 µL. Amplification of the *GH* gene and Sanger sequencing were performed according to the manufacturers' protocols (Evrogen and GenSeq, respectively) (Table 1).

**Table 1:** Amplification conditions for PCR and sequencing.

**Stage**	**PCR amplification conditions**	**Sequencing amplification conditions**
Initial denaturation	95°C, 3 min, 1 cycle	95°C, 1 min, 1 cycle
Denaturation	95°C, 15 s, 30 cycles	95°C, 10 s, 30 cycles
Annealing	63°C, 15 s, 30 cycles	63°C, 10 s, 30 cycles
Elongation	72°C, 30 s, 30 cycles	60°C, 2 min, 30 cycles
Final elongation	72°C, 2 min, 1 cycle	–

PCR = Polymerase chain reaction.

Capillary electrophoresis was performed using a Nanofor 05 genetic analyzer with PDMA-6 sequencing polymer and 1× TAPS sequencing buffer (Syntol). Electrophoresis parameters were established according to the GenSeq kit instructions, considering the capillary length (50 cm), polymer type (PDMA-6), and fragment size. Raw sequencing data in AB1 format were analyzed using Mutation Surveyor v5.2.0 (SoftGenetics, Pennsylvania, USA) (Figures 1 and 2).

**Figure 1:**
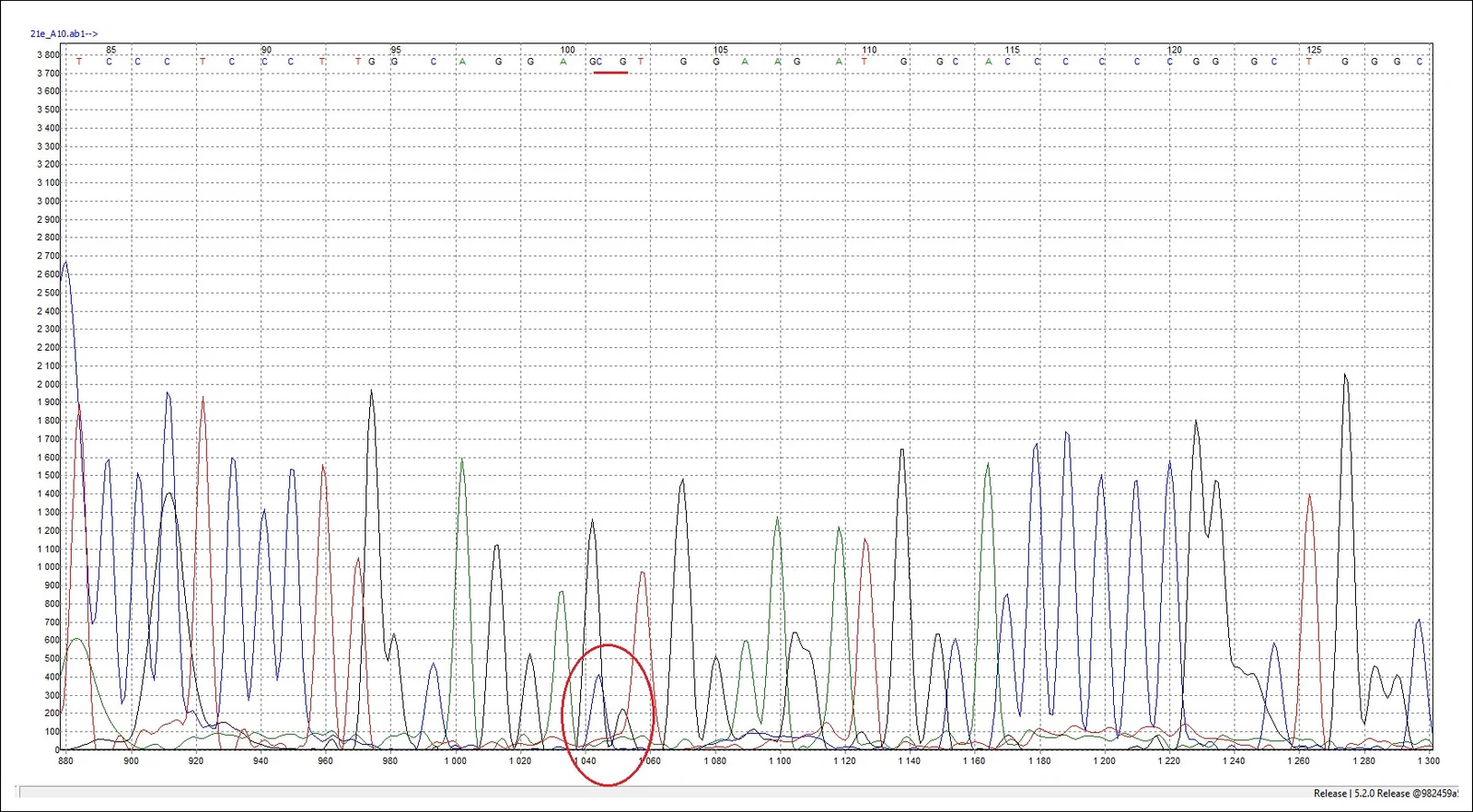
Sanger sequencing electropherogram of the heterozygous LV genotype of the *GH* gene. A heterozygous C/G substitution was detected in exon 5 at chr19:48118256C>G (rs41923484, L127V). The C:G peak ratio was 1.05:1. The variant was confirmed by sequencing in both forward and reverse directions.

### Data collection and laboratory analysis

Blood samples from Kalmyk bull calves (n = 50) were used for genotyping. Blood was collected using vacuum blood collection systems comprising a vacuum syringe-container and specialized needle (Lab-Vac, China). Sampling was performed at the evaluation station in November 2024 (Act No. 5, November 10, 2024), after which the samples were submitted to the Molecular Genetics Laboratory of Kalmyk State University named after B.B. Gorodovikov for analysis.

For physicochemical analysis of beef, *longissimus dorsi* muscle samples were collected from the right half-carcass between the 9th and 11th ribs following controlled slaughter of bull calves representing the two genotypes (n = 16). Samples were collected from 15-month-old bull calves in June 2025 (Act No. 2, June 7, 2025) and provided to Kalmyk State University named after B.B. Gorodovikov for subsequent transport and laboratory analysis.

Assessment of live weight dynamics, calculation of average daily gain, and physicochemical analyses were conducted without knowledge of genotype-group allocation until completion of the primary analyses. Before the experiment, all operators were trained in standardized weighing procedures, with measurements recorded to the nearest 0.1 kg, and in low-stress animal restraint. Interoperator consistency was maintained at a coefficient of variation <2%. Industrial scales of the NAIS brand (LLC «New Automated Measuring Systems», Russia) were calibrated monthly against reference standards, and calibration results were documented. All experimental procedures were performed using standardized checklists with precise timestamps in accordance with the study protocol.

**Figure 2:**
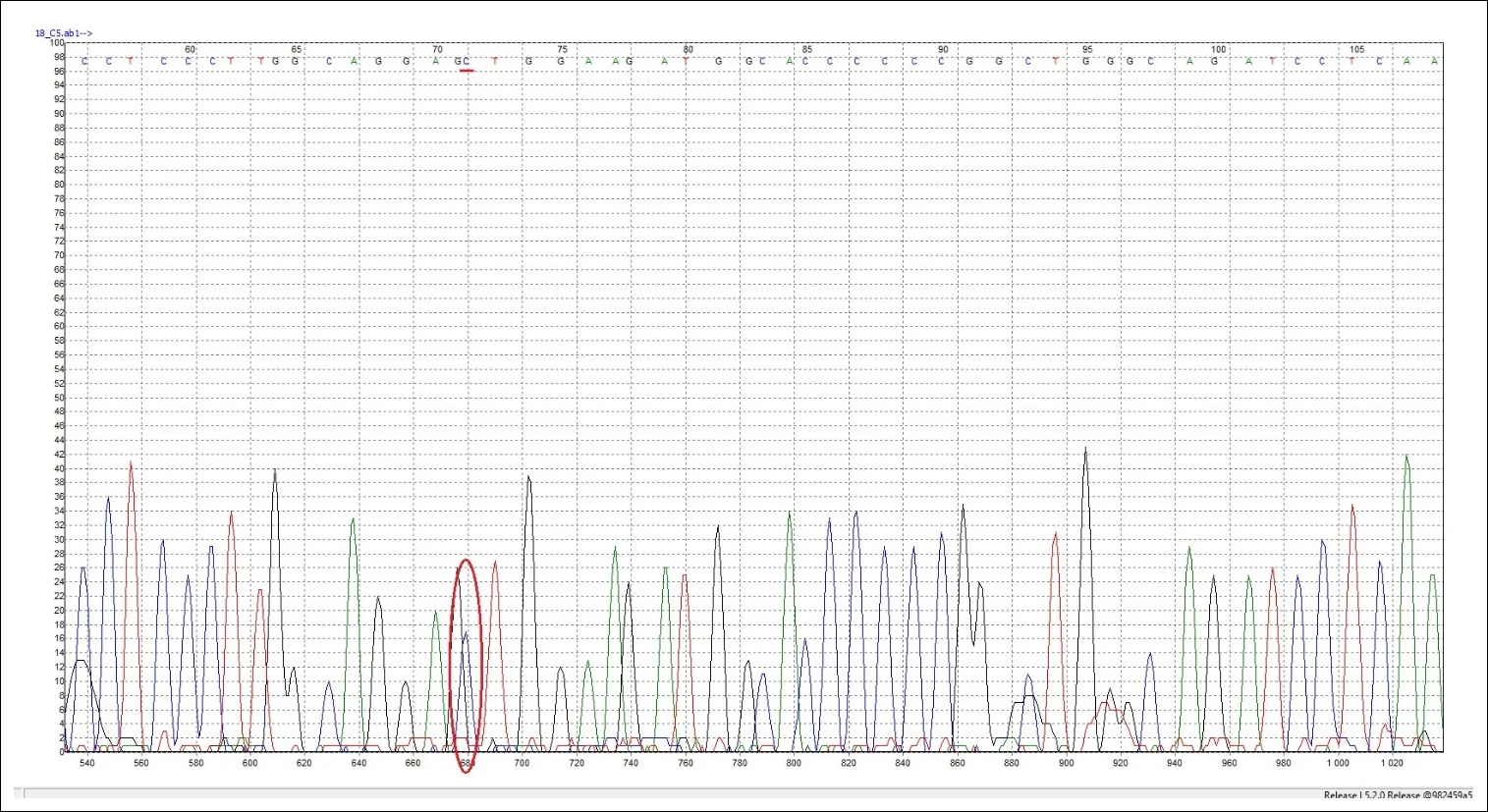
Sanger sequencing electropherogram of the homozygous LL genotype of the *GH* gene (wild type). A single C peak was detected at the variant position in exon 5, chr19:48118256C>G (rs41923484, L127V). The genotype was confirmed by sequencing in both forward and reverse directions.

### Physical and chemical analysis of beef samples

Materials and reagents included 10% neutral buffered formalin as a fixation solution; hematoxylin, eosin, Van Gieson's solution, and Masson's trichrome for histological staining; reagents required for the respective biochemical analyses; standard buffer solutions; and distilled water. Laboratory equipment included an analytical balance with an accuracy of ±0.0001 g, a spectrophotometer with a wavelength range of 200–1000 nm, a pH meter with an accuracy of ±0.01, a texture analyzer with a load capacity of up to 50 kg, a colorimeter based on the CIE 1976 system, a microtome with a section thickness of 5 µm, and ImageJ software for morphometric analysis.

Analyses were performed according to the corresponding GOST standards: GOST 23042-2015 for determination of fat in meat and meat products; GOST 23041-2015 for determination of hydroxyproline; GOST 25011-2017 for determination of protein; GOST 30178-96 for determination of toxic elements in raw materials and food products by atomic absorption spectrometry; GOST 31727-2012 for determination of total ash; GOST 33424-2015 for determination of magnesium by flame atomic absorption spectrometry; GOST 34132-2017 for determination of the amino acid composition of animal proteins; GOST 55573-2013 for determination of calcium by atomic absorption and titrimetric methods; and GOST R 55484-2013 for determination of sodium, potassium, magnesium, and manganese by flame atomic absorption spectrometry.

### Statistical analysis

Intergroup differences were assessed using Welch's t-test with Bonferroni correction to control the Type I error rate. The adjusted significance level was calculated as α_adj = 0.05/k, where k ranged from 3 to 11 depending on the number of parameters analyzed [32]. Effect sizes were estimated using Cohen's d [33]. For selected analyses, 95% confidence intervals (CIs) for effect sizes were additionally calculated using the noncentral t-distribution [34]. Data distribution was assessed for normality using the Shapiro–Wilk test. 

Statistical analyses and data visualization were performed using Python v3.12/3.13 (python.org), pandas v2.2.3 (pydata.org), SciPy v1.14.1 (scipy.org), Matplotlib v3.9.3 (matplotlib.org), and Seaborn v0.13.2 (pydata.org). Results are presented as mean ± standard deviation (M ± SD).

### Quality assessment and risk-of-bias

The methodological quality of the study and risk-of-bias were assessed using the Systematic Review Center for Laboratory Animal Experimentation (SYRCLE) risk-of-bias tool in accordance with the Animal Research: Reporting of *In Vivo* Experiments (ARRIVE) 2.0 guidelines [35, 36]. The assessment covered the principal domains of potential bias, with each domain classified as having a low, moderate, or unclear risk of bias.

The study design and predefined endpoints, including growth performance, slaughter traits, and meat-quality characteristics, were established before initiation of the experiment. Animals were classified according to genotype (LL, n = 37; LV, n = 13) based on objective genotyping results. The genotype groups were comparable in age and initial live weight, and allocation to housing sections was performed using stratified randomization by farm of origin.

Detection bias was minimized through blinding. Personnel responsible for measurements and laboratory analyses were unaware of the animals' genotypes during primary data collection and analysis. Calibrated measurement instruments and standardized experimental procedures were used throughout the study. Attrition bias was considered low because all animals completed the experimental protocol and complete data were available for the predefined outcomes.

Performance bias was minimized by standardizing major husbandry conditions, including diet, housing area (75 m² per section), ventilation, straw bedding, and daily cleaning, together with a 14-day adaptation period. Nevertheless, complete control of microenvironmental conditions was not possible. Potential sources of bias included variations in temperature and humidity, possible fluctuations in feed quality, and microenvironmental gradients related to proximity to feeders and ventilation openings. These factors were not incorporated into the statistical models.

The statistical approach incorporated tests appropriate to the data characteristics, along with Bonferroni correction for multiple comparisons and Cohen's d to estimate effect magnitude. The overall sample comprised 50 animals, with eight animals from each genotype selected for meat-quality assessment. The latter sample size was determined a priori to provide 80% statistical power at α_adj = 0.05 based on an expected Cohen's d > 0.8. The final risk-of-bias assessment is presented in Table 2.

**Table 2:** Assessment of risk-of-bias according to the SYRCLE tool.

**SYRCLE domain**	**Risk-of-bias assessment**	**Justification**
Selection bias	Low	Genotype distribution reflected the naturally occurring allele frequencies in the study population; groups were comparable in age and initial live weight, and genotyping was performed objectively.
Performance bias	Moderate	Feeding and housing conditions were standardized; however, uncontrolled microenvironmental factors could have contributed to confounding.
Detection bias	Low	Blinding was applied during measurements and primary analyses, and calibrated instruments and standardized measurement protocols were used.
Attrition bias	Low	All animals completed the study, and no data were missing for the key outcomes.
Reporting bias	Low	The study was designed and reported in accordance with ARRIVE guidelines, and the predefined outcomes were reported.

ARRIVE = Animal Research: Reporting of *In Vivo* Experiments; SYRCLE = Systematic Review Center for Laboratory Animal Experimentation.

Overall, the study was assessed as having a low risk-of-bias in most domains. The moderate risk of performance bias reflected the inability to completely control microenvironmental conditions within the housing sections. These potential sources of bias were considered when interpreting the findings, while effect-size estimation complemented significance testing by quantifying the magnitude of the observed differences.

## RESULTS

### Genotyping

The *GH* c.2141C>G polymorphism in the bull calves (n = 50) was represented by the L and V alleles, with frequencies of 87% and 13%, respectively. The LL and LV genotype frequencies were 74% and 26%, respectively (Figure 3). The VV genotype was not detected in the study population.

The absence of the VV genotype was consistent with the low frequency of the V allele and the correspondingly low expected frequency of VV homozygotes. The V allele was therefore observed exclusively in the heterozygous state in the study population.

**Figure 3:**
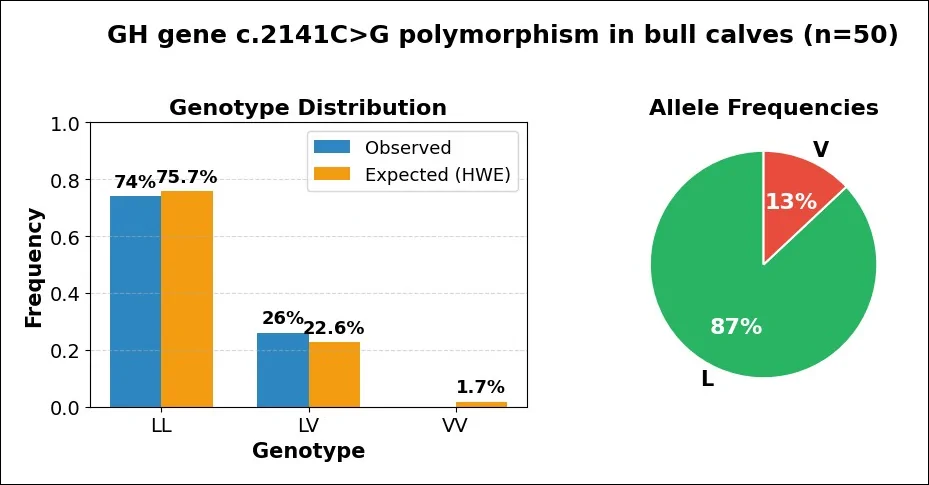
Hardy–Weinberg equilibrium analysis of the distribution of LL and LV genotypes in bull calves (n = 50) using the χ² test.

### Growth performance according to genotype

Live weight was evaluated in LL and LV bull calves at 8, 12, and 15 months of age (Figure 4). LV calves had numerically higher live weights than LL calves at all three ages. The differences between the LV and LL groups were 4.45 ± 3.5 kg at 8 months, 20.5 ± 6.8 kg at 12 months, and 25.9 ± 6.2 kg at 15 months. However, after correction for multiple comparisons, none of the differences were statistically significant. Cohen's d values ranged from −0.38 to −0.71, indicating moderate-to-large effect magnitudes despite the absence of statistical significance.

**Figure 4:**
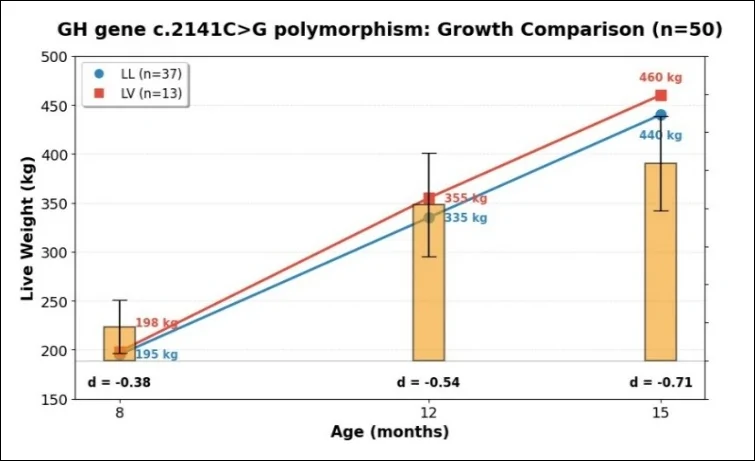
Live weight of LL and LV bull calves at different ages (n = 50). Differences between genotype groups were assessed using Welch's t-test with Bonferroni correction (α_corr = 0.0167). No statistically significant differences were detected at any age (p > 0.0167). Effect sizes were estimated using Cohen's d.

Growth intensity was further evaluated by comparing average daily gain between genotypes during the 8–12-, 12–15-, and 8–15-month periods (Figure 5). LV calves had numerically higher average daily gains than LL calves during all evaluated periods. The difference was 11.5% during the 8–12-month period (122 days), 5.1% during the 12–15-month period, and 8.8% over the entire 8–15-month period. None of these differences were statistically significant (p > 0.05). Cohen's d values ranged from −0.20 to −0.58, corresponding to small-to-moderate effect magnitudes. Thus, the present data did not establish a statistically significant association between genotype and average daily gain.

### Slaughter traits according to genotype

Slaughter traits were evaluated at 15 months of age in eight animals from each genotype-group (Figure 6). Statistically significant differences between the LL and LV groups were detected for carcass weight and internal fat weight (p < 0.05), whereas dressing percentage did not differ significantly between genotypes (p > 0.05). The largest effect-size was observed for internal fat weight (Cohen's d = 3.53; 95% CI = 1.58–5.48), followed by carcass weight (d = 2.28; 95% CI = 1.02–3.54). In contrast, the effect-size for dressing percentage was small (d = 0.14), and its 95% CI included zero.

**Figure 5:**
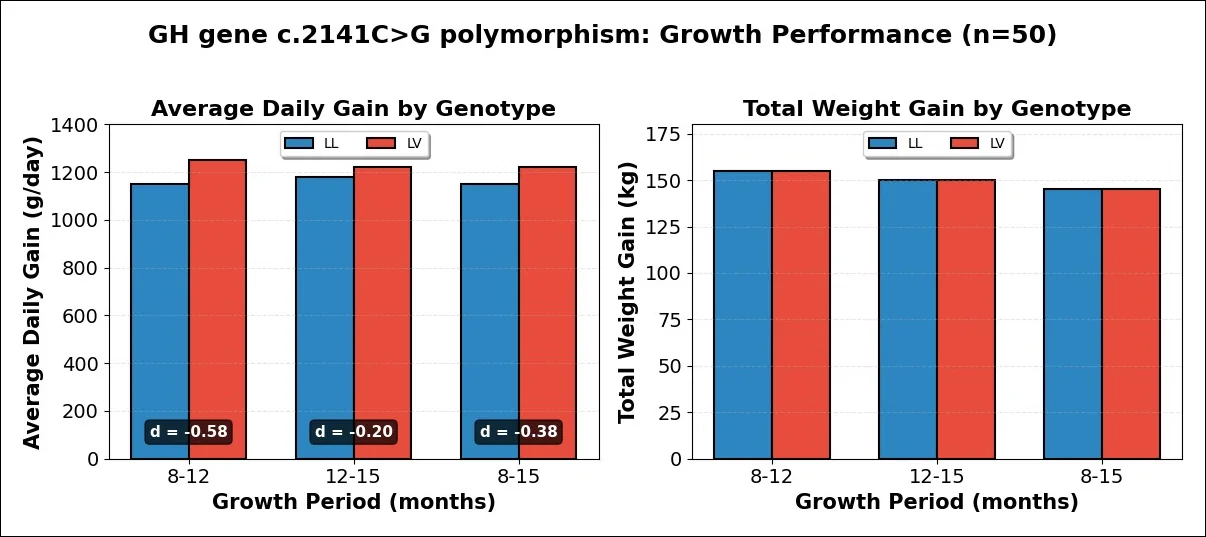
Average daily gain of LL and LV bull calves during the 8–12-, 12–15-, and 8–15-month age periods. Values are presented as mean ± standard deviation (M ± SD). Intergenotype differences were assessed using Student's t-test or the Mann–Whitney U test according to the assumptions of normality and homoscedasticity. No statistically significant differences were detected between genotype groups during any age period (p > 0.05). Cohen's d ranged from −0.20 to −0.58.

**Figure 6:**
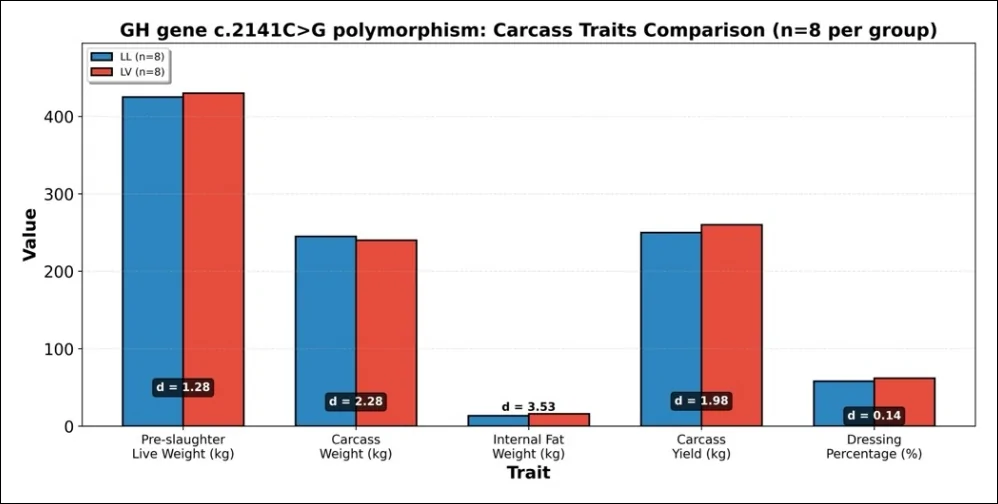
Meat productivity traits of LL and LV bull calves at 15 months of age (n = 8 per genotype). Carcass weight, internal fat weight, and dressing percentage are presented as mean ± standard deviation (M ± SD). Group comparisons were performed using Student's t-test or the Mann–Whitney U test according to the assumptions of normality (Shapiro–Wilk test) and homoscedasticity (Levene's test). Significant differences were detected for carcass weight and internal fat weight (p < 0.05; Cohen's d = 2.28–3.53), whereas dressing percentage did not differ significantly between genotypes (p > 0.05; d = 0.14).

### Chemical composition of beef

The chemical composition of beef differed significantly between the LL and LV genotypes for moisture, protein, fat, and ash contents (Figure 7). Beef from LV calves contained, on average, 1.01% more protein and 0.23% more ash than beef from LL calves, whereas LL samples contained 0.50% more fat and 0.40% more moisture. The p-values for these four parameters ranged from <0.001 to 0.0013. Cohen's d values ranged from 1.25 to 1.47, and the corresponding 95% CIs for the mean differences did not include zero, indicating large effect magnitudes.

### Essential amino acid composition and amino acid score

Essential amino acid composition and amino acid scores were evaluated to characterize the protein quality of beef from the two genotype groups (Figure 8). Mean essential amino acid concentrations were consistently higher in LV than in LL samples, with the largest numerical differences observed for tryptophan (+30%) and histidine (+11%) (Figure 8A).

The amino acid score was calculated as the ratio of the mass fraction of each essential amino acid to that of the reference protein. The analyzed beef samples met the FAO/WHO recommended essential amino acid level of at least 29 g/100 g protein [37]. Amino acid scores exceeded 100% for seven of the nine evaluated amino acids, with valine and isoleucine as the exceptions. High mean amino acid scores were observed for tryptophan and histidine in both genotype groups; however, the differences were not statistically significant after Bonferroni correction (p > 0.0045). Cohen's d values of up to 0.7–0.9 were observed for these amino acids.

**Figure 7:**
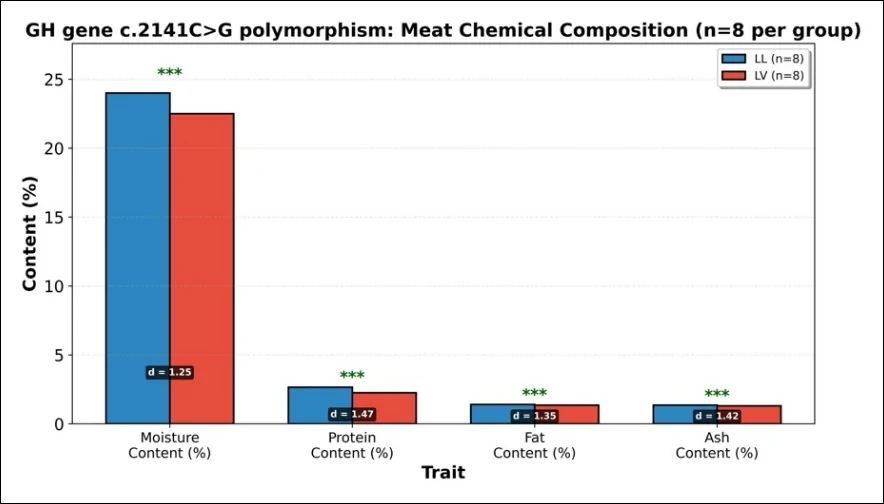
Moisture, protein, fat, and ash contents of beef from LL and LV bull calves (n = 8 per genotype). Data are presented as mean ± standard deviation (M ± SD). Differences between genotype groups were evaluated using Welch's t-test. The p-values ranged from <0.001 to 0.0013, and Cohen's d values ranged from 1.25 to 1.47. The 95% CIs for the mean differences did not include zero.

**Figure 8:**
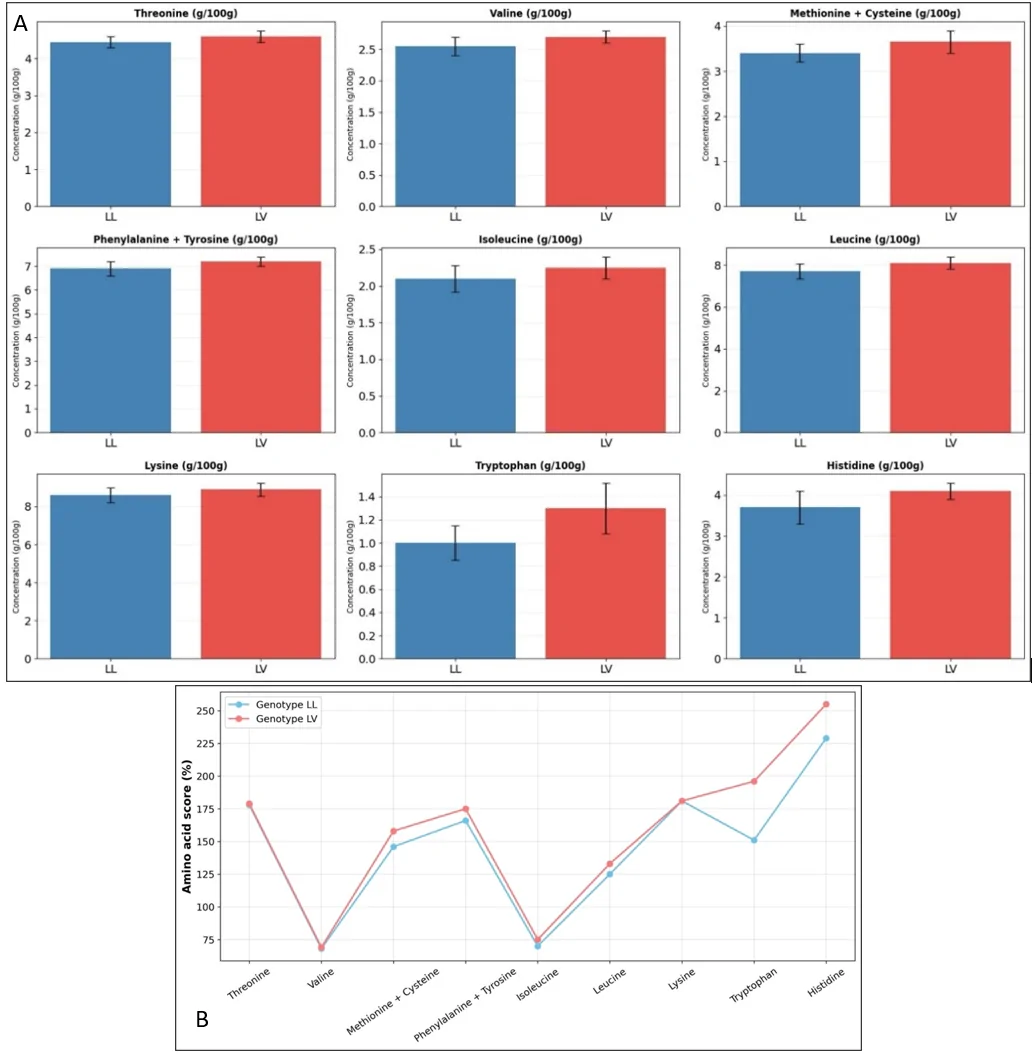
Essential amino acid composition and amino acid scores of beef samples from LL and LV bull calves (n = 16). (A) Essential amino acid content. (B) Amino acid score. Data are presented as mean ± standard deviation (M ± SD). Group comparisons were performed using Welch's t-test with Bonferroni correction (α_corr = 0.0045). *Methionine plus cysteine and phenylalanine plus tyrosine were evaluated as combined values because cysteine can be synthesized from methionine and tyrosine from phenylalanine.

### Non-essential amino acid composition

No statistically significant genotype-associated differences were detected in the non-essential amino acid composition of the beef samples after correction for multiple comparisons (p > 0.0063) (Figure 9). Moderate effect sizes (Cohen's d up to 0.6–0.8) were observed for glycine and hydroxyproline, with the largest numerical differences occurring for these amino acids. Glutamic and aspartic acids were present at relatively high concentrations in samples from both genotype groups.

**Figure 9:**
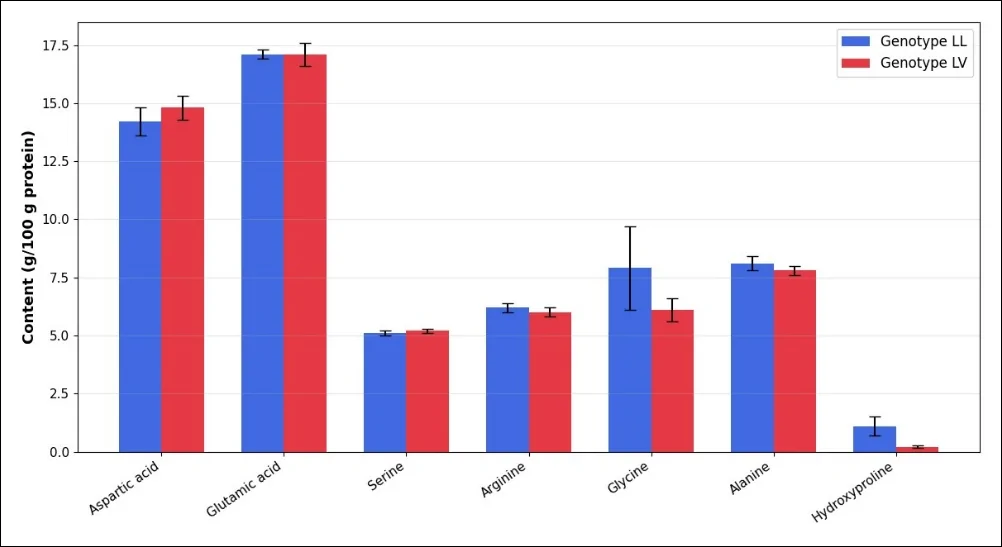
Glycine, hydroxyproline, glutamic acid, and aspartic acid contents of beef from LL and LV bull calves (n = 8 per genotype). Data are presented as mean ± standard deviation (M ± SD). Group comparisons were performed using Student's t-test or the Mann–Whitney U test according to the assumptions of normality (Shapiro–Wilk test) and homoscedasticity (Levene's test).

The summary analysis of essential, non-essential, and total amino acid contents and their relative proportions is presented in Table 3.

**Table 3:** Amino acid profile of beef from LL and LV bull calves: Mean differences, 95% CIs, Cohen's d, and p-values (n = 16).

**Parameter**	**Mean difference (LV − LL)**	**95% CI**	**Cohen's d**	**p-value**
ΣEAA, g/100 g protein	+1.83	−2.56–6.22	0.788	0.407
ΣNEAA, g/100 g protein	−1.07	−4.89–2.75	0.555	0.542
Σtotal AA, g/100 g protein	+0.77	−1.16–2.70	1.606	0.184
ΣEAA/ΣNEAA	+0.05	−0.09–0.19	0.705	0.453
ΣEAA, proportion of Σtotal AA	+0.01	−0.02–0.04	0.851	0.381

AA = Amino acids; CI = confidence interval; EAA = essential amino acids; NEAA = non-essential amino acids. Mean difference = mean (LV) − mean (LL). Statistical significance was evaluated against the Bonferroni-adjusted threshold of α_adj = 0.01.

The LV genotype had numerically higher total EAA content (42.28 vs. 40.45 g/100 g protein), total AA content (99.79 vs. 99.02 g/100 g protein), EAA/NEAA ratio (0.74 vs. 0.69), and proportion of EAA in total AA (0.42 vs. 0.41) than the LL genotype. However, none of the parameters presented in Table 3 reached the Bonferroni-adjusted threshold for statistical significance (all p > 0.01). Therefore, these findings indicate numerical genotype-associated differences in the amino acid profile but do not provide statistical evidence of differences for these aggregate amino acid measures under the present study conditions.

## DISCUSSION

The present study investigated the association of the *GH* c.2141C>G polymorphism with meat productivity and beef quality in Kalmyk bull calves. The findings generally support previous evidence suggesting that the LV genotype may be associated with favorable meat productivity traits. Dzhulamanov and Gerasimov [13] reported greater growth intensity during fattening in heterozygous animals than in animals with the LL genotype. In their study, LV animals had a 7.6% higher preslaughter live weight than LL animals, although the difference was not statistically significant (p > 0.05), and a 13.61% higher carcass weight (p < 0.05). LV bull calves also had 4.4% greater internal fat accumulation than LL calves, although this difference was not statistically significant (p > 0.05). These observations are consistent with the direction of the differences observed in the present study, particularly for carcass weight and internal fat weight.

In the present study, LV calves showed numerically higher live weights and average daily gains across the evaluated age periods, although these differences did not reach statistical significance. In contrast, statistically significant genotype-associated differences were detected for several slaughter traits among the animals selected for slaughter (n = 8 per genotype). The most pronounced effects were observed for internal fat weight (Cohen's d = 3.53) and carcass weight (d = 2.28), indicating large differences between the genotype groups. Dressing percentage, however, did not differ significantly between LL and LV animals. These results indicate that the association of the *GH* c.2141C>G polymorphism with slaughter characteristics was more pronounced than its association with longitudinal growth performance under the conditions of the present study. Nevertheless, interpretation should account for the relatively small number of animals evaluated for slaughter and the unequal genotype frequencies in the overall study population.

The chemical composition of beef also differed between genotype groups. Moisture, protein, fat, and ash contents differed significantly between LL and LV animals (p < 0.001–0.0013), with Cohen's d values ranging from 1.25 to 1.47 and 95% CIs for the mean differences not including zero. Beef from LV animals contained 1.01% more protein and 0.23% more ash, whereas beef from LL animals contained 0.50% more fat and 0.40% more moisture. These findings provide evidence that variation at the *GH* c.2141C>G locus may also be associated with differences in the chemical composition of beef rather than exclusively with growth and carcass characteristics. The greater fat content observed in LL animals is partially consistent with the findings of Safonova [38], who similarly reported more pronounced fat accumulation in animals with the LL genotype than in those with the LV genotype.

Not all previous findings, however, support an advantage of the LV genotype. Sedykh *et al.* [39] reported that Hereford and Limousin bull calves with the LL genotype had higher preslaughter live weights than LV and VV animals, by 4.95% and 4.18%, respectively (p < 0.01). This differs from the numerical advantage of LV animals observed in the present study. Differences among studies may reflect breed-specific genetic backgrounds, husbandry and feeding conditions, genotype frequencies, sample sizes, and experimental designs. Thus, the phenotypic association of the *GH* c.2141C>G polymorphism may not be uniform across cattle populations, emphasizing the importance of evaluating candidate genetic markers within the specific breed and production environment in which they are intended to be applied.

Evidence concerning the relationship between the *GH* c.2141C>G polymorphism and the chemical characteristics of beef remains comparatively limited. Although the present results identified significant genotype-associated differences in the principal chemical components of beef, the relatively small number of animals analyzed for meat quality restricts the extent to which these findings can be generalized. Larger studies involving independent Kalmyk cattle populations and, where possible, multiple production environments are therefore required to determine the reproducibility and magnitude of these associations. Such studies would help clarify whether the *GH* c.2141C>G polymorphism has sufficient and consistent predictive value to contribute to marker-assisted selection for meat productivity and quality traits in Kalmyk cattle.

## CONCLUSION

This study demonstrated that the *GH* c.2141C>G polymorphism was associated with distinct growth, slaughter, and beef quality characteristics in Kalmyk bull calves. Although LV animals showed consistently higher live weights and average daily gains than LL animals across the evaluated age periods, these differences were not statistically significant. In contrast, genotype-associated differences were more pronounced for slaughter traits. LV animals had greater carcass weight and internal fat weight, with large effect sizes (Cohen's d = 2.28 and 3.53, respectively), whereas dressing percentage did not differ significantly between genotypes. Beef chemical composition also differed significantly between LL and LV animals, with LV samples containing more protein and ash and LL samples containing more fat and moisture. Cohen's d values for these chemical traits ranged from 1.25 to 1.47, supporting substantial differences between genotype groups. Differences in individual and aggregate amino acid characteristics were predominantly numerical and did not remain statistically significant after correction for multiple comparisons.

The findings indicate that the *GH* c.2141C>G polymorphism may have greater relevance to carcass characteristics and beef chemical composition than to growth rate alone in Kalmyk cattle. In particular, the association of the LV genotype with greater carcass weight and higher protein content may be relevant to breeding programs targeting improved meat yield and nutritional characteristics. However, the polymorphism should not yet be used as an independent selection criterion, and its potential application would be more appropriate as one component of a broader marker-assisted selection strategy incorporating phenotypic performance and additional genetic markers.

A major strength of this study was the integrated assessment of genotype in relation to longitudinal growth performance, slaughter characteristics, and laboratory-determined beef composition within the same experi-mental population. Standardized housing and feeding conditions, objective genotyping, blinded measurement and laboratory assessment, calibration of measurement equipment, correction for multiple comparisons, and complementary estimation of effect sizes strengthened the interpretation of the observed genotype-associated differences. Including effect-size estimates was particularly important because it enabled assessment of the magnitude of differences beyond reliance on statistical significance alone.

The principal limitation was the relatively small number of animals used for slaughter and meat-quality assessment (eight animals per genotype), which restricts the precision and generalizability of the estimates. In addition, the natural genotype distribution resulted in unequal numbers of LL and LV animals in the overall population, and the VV genotype was not detected. Consequently, the study could not evaluate the full range of genotype effects at the *GH* c.2141C>G locus. The study was also conducted in a single breed and production environment, while uncontrolled microenvironmental factors, including variation in temperature, humidity, feed quality, and proximity to feeders or ventilation, may have contributed to residual variability. These considerations are particularly relevant for traits that showed moderate or large effect sizes but did not reach statistical significance.

Future studies should therefore include larger and independently sampled Kalmyk cattle populations, incorporate sufficient numbers of all three genotypes where biologically available, and evaluate the reproducibility of the observed associations across farms, feeding systems, and environmental conditions. Additional work should also examine interactions between *GH* c.2141C>G and other candidate loci or genomic markers associated with growth, carcass composition, and meat quality. Validation in larger cohorts would help determine whether the observed effects are sufficiently stable and predictive for incorporation into practical breeding programs.

Overall, the *GH* c.2141C>G polymorphism showed a stronger association with slaughter traits and beef chemical composition than with growth dynamics in the studied Kalmyk bull calves. The LV genotype was associated with favorable carcass characteristics and higher protein and ash contents, whereas the LL genotype was associated with higher fat and moisture contents. These findings expand the available breed-specific evidence for Kalmyk cattle and support further evaluation of the *GH* c.2141C>G polymorphism as a potential component of marker-assisted selection. Nevertheless, larger validation studies are required before this polymorphism can be recommended for routine breeding decisions.

## DATA AVAILABILITY

The datasets generated and/or analyzed during the current study are not publicly available due to institutional restrictions but can be obtained from the corresponding author upon reasonable request.

## GENERATIVE AI DECLARATION

No generative artificial intelligence tools were used in the design, execution, analysis, or writing of this study. All data, analyses, interpretations, and text were produced solely by the authors.

## AUTHORS’ CONTRIBUTIONS

NC and ZB: Designed and conducted the study and drafted and edited the manuscript, project supervision and article final editing. VM and AK: collected samples and conducted phenotype scoring. AU and VU: conducted genetic investigation. NR and AS: carried out organoleptic and biochemical investigation. All authors have read and approved the final version of the manuscript.
